# Day type and heatstroke-related ambulance transports in Tokyo, Japan: a time-series study

**DOI:** 10.1265/ehpm.26-00077

**Published:** 2026-08-07

**Authors:** Yuji Nishiwaki, Sachie Mori, Takehiro Michikawa

**Affiliations:** 1 Department of Environmental and Occupational Health, School of Medicine, Toho University, 5-21-16 Omori-Nishi, Ota, Tokyo 143-8540, Japan; 2 Department of Community Well-Being, School of Medicine, Toho University, 5-21-16 Omori-Nishi, Ota, Tokyo 143-8540, Japan

**Keywords:** Heatstroke, Ambulance transport, Day type, Time-series analysis, Behavioural factors

## Abstract

**Background:**

Heatstroke is a major public health problem during summer, and meteorological conditions, particularly wet bulb globe temperature (WBGT), strongly influence its occurrence. However, even under similar heat conditions, heatstroke risk may vary according to behavioural patterns on weekdays, weekends/holidays, and post-holiday days. This study aimed to examine whether heatstroke-related ambulance transports in Tokyo vary according to day type after adjusting for WBGT.

**Methods:**

We conducted a time-series analysis using daily heatstroke-related ambulance transports in Tokyo, Japan, during the warm season (May–September) from 2015 to 2025. The days were classified as weekday, weekend/holiday, or post-holiday. The WBGT data for seven locations in Tokyo were used, and the highest daily maximum WBGT served as the heat exposure indicator. Modified Poisson regression models were used to estimate risk ratios (RRs) and 95% confidence intervals (CIs) according to day type, adjusting for the WBGT category at lags 0 and 1 and the calendar year. The same models were applied by age group, severity, and place of occurrence. Four sensitivity analyses, including the use of alternative heat exposure indicators and exclusion of the coronavirus disease 2019 period, were conducted.

**Results:**

After adjusting for WBGT and calendar year, the occurrence of heatstroke-related ambulance transports differed according to day type. Overall, ambulance transports tended to be more frequent on post-holiday days than on weekdays (RR, 1.08; 95% CI, 0.97–1.22). Among adults aged ≥65 years, transports were more frequent on post-holiday days (RR, 1.13; 95% CI, 1.00–1.28) and less frequent on weekends/holidays (RR, 0.87; 95% CI, 0.79–0.96). Among individuals aged <18 years, transports were markedly more frequent on weekends/holidays (RR, 1.80; 95% CI, 1.61–2.03). Weekend/holiday increases were also observed in mild cases and in public indoor and outdoor spaces and educational institutions, whereas cases in residences and workplaces were less frequent. Sensitivity analyses yielded similar results.

**Conclusions:**

Heatstroke-related ambulance transports in Tokyo varied according to day type even after adjusting for WBGT, with distinct patterns across age groups, severity categories, and places of occurrence. Weekly behavioural patterns may contribute to heatstroke risk and warrant consideration in preventive strategies.

**Supplementary information:**

The online version contains supplementary material available at https://doi.org/10.1265/ehpm.26-00077.

## Graphical Abstract

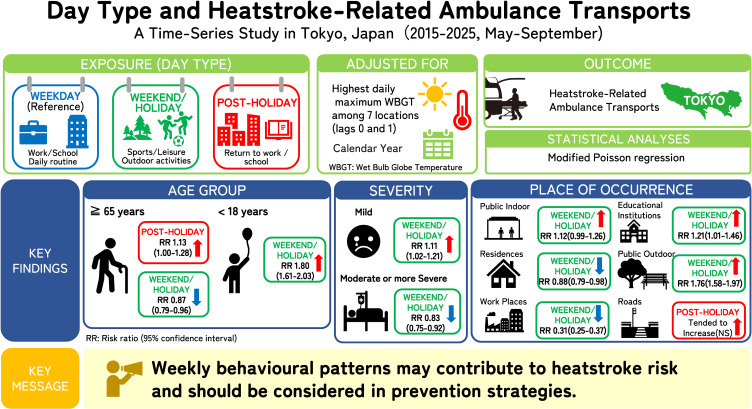

## Background

In recent years, human-induced climate change has caused a significant rise in the Earth’s average temperature, leading to an increase in the frequency and intensity of extreme heat events worldwide [1, 2]. Extreme heat poses serious risks to human health [3]. Heatstroke is one of the most common health problems caused by exposure to high temperatures and is a major public health issue during summer [4, 5]. In Japan, the number of heatstroke-related ambulance transports is projected to increase under climate change and population aging scenarios [6].

Several epidemiological studies in Japan have demonstrated strong associations between wet bulb globe temperature (WBGT) and heatstroke-related ambulance transports [7–9]. WBGT integrates air temperature, humidity, and radiant heat and is widely used as an indicator of environmental heat stress [10]. Thus, meteorological conditions are major determinants of heatstroke risk. However, even under similar heat conditions, heatstroke occurrence may vary depending on differences in people’s behaviours and activity patterns.

Activities, such as outdoor work, sports, and recreation, vary according to the day of the week and public holidays. In particular, on the day immediately following holidays, behavioural patterns may substantially change as work and school activities resume. Understanding these temporal patterns from a public health perspective may help improve risk communication and prevention strategies. For example, by intensively disseminating heatstroke prevention messages before weekends or immediately after long holidays, it may be possible to effectively reach higher-risk groups during specific periods. However, to our knowledge, no previous studies have examined whether heatstroke-related ambulance transports vary according to day type after adjusting for WBGT.

Tokyo is Japan’s largest metropolitan area, and during summer, there are numerous emergency transports due to heatstroke. Furthermore, because WBGT data are available for multiple locations within the city, it is well-suited for assessing heat exposure on an urban scale. Therefore, this study aimed to examine whether heatstroke-related ambulance transports in Tokyo differ according to day type using a time-series analysis adjusted for WBGT. To further characterise these patterns, we applied the same analytical approach to daily ambulance transports by age group, severity, and place of occurrence.

## Methods

This study was a time-series analysis in which the outcome was the daily number of heatstroke-related ambulance transports in Tokyo.

### Exposure assessment

The main exposure variable was day type. Days were categorised into three groups: weekday, weekend/holiday, and post-holiday. Weekend/holiday included Saturdays, Sundays, and national public holidays. Post-holiday was defined as the first weekday immediately following weekends or national public holidays (e.g. Monday after a weekend or the next working day after a public holiday). Weekdays were defined as weekdays excluding post-holiday days and were treated as a single category, as preliminary analyses showed no substantial differences in ambulance transports across individual weekdays.

### Outcome

The outcome was the daily number of heatstroke-related ambulance transports in Tokyo, Japan. In Japan, ambulance services are available free of charge at all times. The data were obtained from publicly available records released by the Fire and Disaster Management Agency of the Ministry of Internal Affairs and Communications [11]. The study period covered 2015–2025 (2025 data were provisional). The analyses were restricted to the warm season (May to September) of each year. As data for May 2020 were unavailable, only June–September were used for that year. The dataset includes the total number of heatstroke-related ambulance transports as well as counts by age group, severity, and place of occurrence, all of which were classified and reported in the original dataset. In the original dataset, age was categorised into five groups (<28 days, 28 days–6 years, 7–17 years, 18–64 years, and ≥65 years). Because the numbers of cases in the two youngest age groups were small, age groups were reclassified into three categories for analysis: <18 years, 18–64 years, and ≥65 years. Severity was categorised into two groups: mild and moderate or more severe cases. For place of occurrence, categories with very small numbers were excluded, and the remaining locations were grouped into six categories: public indoor spaces, residences, workplaces (e.g. construction sites, factories, and workshops), educational institutions, public outdoor spaces (e.g. stadiums, outdoor car parks, outdoor concert venues, and outdoor railway platforms), and roads.

### Covariates

Covariates included WBGT category and calendar year. WBGT data for seven locations in Tokyo (Bunkyo, Nerima, Hachioji, Fuchu, Edogawa-Rinkai, Ogouchi, and Ome), excluding island areas, are publicly available on the Ministry of the Environment website [12]. The daily maximum WBGT was calculated for each location, and the highest value across the seven locations was used as an indicator of heat exposure for Tokyo, as heatstroke risk increases under higher heat stress. WBGT was selected because it is widely used for heatstroke prevention and heat alerts in Japan. WBGT was categorised into five groups (<25.0, 25.0–27.9, 28.0–30.9, 31.0–32.9, and ≥33.0 °C) based on the heat illness prevention guidelines of the Japan Sports Association [13] and the heatstroke alert threshold issued by the Ministry of the Environment [14].

### Statistical analyses

First, descriptive statistics of heatstroke-related ambulance transports and WBGT were summarised according to day type. Next, the association between day type and heatstroke-related ambulance transports was examined using modified Poisson regression models. Weekdays were used as the reference category, and risk ratios (RRs) and 95% confidence intervals (CIs) were calculated. The models were adjusted for the WBGT category based on the maximum daily WBGT across the seven locations and calendar years. To account for the delayed effects of heat exposure, WBGT categories for the same day (lag 0) and previous day (lag 1) were included in the models. Models including lag 2 were also examined, but results were similar; therefore, lags 0 and 1 were used. The same models were fitted separately for ambulance transports by age group, severity, and place of occurrence. Four sensitivity analyses were performed. First, the exposure indicator was defined using the mean of the daily maximum WBGT across locations instead of the highest value across locations. Second, daily mean air temperature in Kitanomaru, Tokyo [15], was used instead of WBGT and categorised as in the main analysis, with lag 0 and lag 1 temperature categories included in the models. Third, the highest daily maximum WBGT at lag 0 and lag 1 were included as continuous covariates. Fourth, to minimise the influence of changes in human mobility during the coronavirus disease 2019 (COVID-19) pandemic, analyses excluding data from 2020 to 2022 were performed. In Japan, behavioural restrictions related to COVID-19 were lifted in May 2023 [16]. Therefore, data from 2020 to 2022 were excluded.

All statistical analyses were performed using Stata version 19 (StataCorp, College Station, TX, USA), and a P value < 0.05 was considered statistically significant.

This study used publicly available aggregated data and did not involve individual-level information. Therefore, ethical approval was not required.

## Results

Table 1 summarises the distribution of ambulance transports and WBGT according to day type. The proportion of days with a maximum WBGT ≥ 33 °C was slightly higher on post-holiday days, although the difference was not statistically significant (χ^2^ test, P = 0.45).

**Table 1:** Characteristics of heatstroke-related ambulance transports and WBGT conditions according to day type

		**Weekday**	**Post-holiday**	**Weekend/holiday^a^**
**Number of days**		877	255	520
**Ambulance transports per day**				
Total	Mean (median, IQR)	38.8 (12, 3–49)	43.7 (11, 3–66)	37.6 (11.5, 3–50)
Age groups				
Age ≥65	Mean (median, IQR)	20.8 (7, 1–24)	24.5 (6, 1–35)	17.7 (5, 1–23)
Age 18–64	Mean (median, IQR)	15.4 (4, 1–20)	16.3 (4, 1–21)	15.4 (5, 1–20)
Age <18	Mean (median, IQR)	2.5 (1, 0–3)	2.9 (1, 0–4)	4.5 (2, 0–6)
Severity				
Moderate or worse	Mean (median, IQR)	15.9 (5, 1–19)	17.9 (4, 1–25)	12.8 (4, 1–16.5)
Mild	Mean (median, IQR)	22.9 (8, 2–29)	25.8 (6, 1–38)	24.8 (8, 2–33.5)
Locations				
Public (indoor)	Mean (median, IQR)	6.2 (2, 0–7)	7.1 (2, 0–9)	6.8 (2, 0–9)
Residences	Mean (median, IQR)	16.8 (6, 1–22)	18.4 (6, 1–24)	14.7 (4, 1–19)
Workplaces	Mean (median, IQR)	3.4 (1, 0–4)	3.1 (1, 0–4)	1.0 (0, 0–1)
Educational institutions	Mean (median, IQR)	1.7 (1, 0–2)	1.6 (1, 0–2)	2.0 (1, 0–2)
Public (outdoor)	Mean (median, IQR)	3.5 (1, 0–5)	4.1 (1, 0–6)	6.1 (2, 0–8)
Road	Mean (median, IQR)	10.4 (3, 0–13)	12.3 (2, 0–18)	9.2 (2, 0–13)
**WBGT**				
Maximum of max WBGT	Mean, SD	27.7, 4.3	27.6, 4.5	27.6, 4.3
	Days with ≥31 °C, n (%)	270 (30.8)	76 (29.8)	149 (28.7)
	Days with ≥33 °C, n (%)	87 (9.9)	32 (12.6)	58 (11.2)
Average of max WBGT	Mean, SD	26.3, 4.2	26.3, 4.5	26.2, 4.2
	Days with ≥31 °C, n (%)	159 (18.1)	48 (18.8)	87 (16.7)
	Days with ≥33 °C, n (%)	3 (0.3)	5 (2.0)	8 (1.5)

WBGT: wet bulb globe temperature; IQR: interquartile range; SD: standard deviation

^a^Day types were defined as post-holiday (Mondays and the day following a public holiday), weekday, and weekend/holiday (Saturdays, Sundays, and public holidays).

**Fig. 1:**
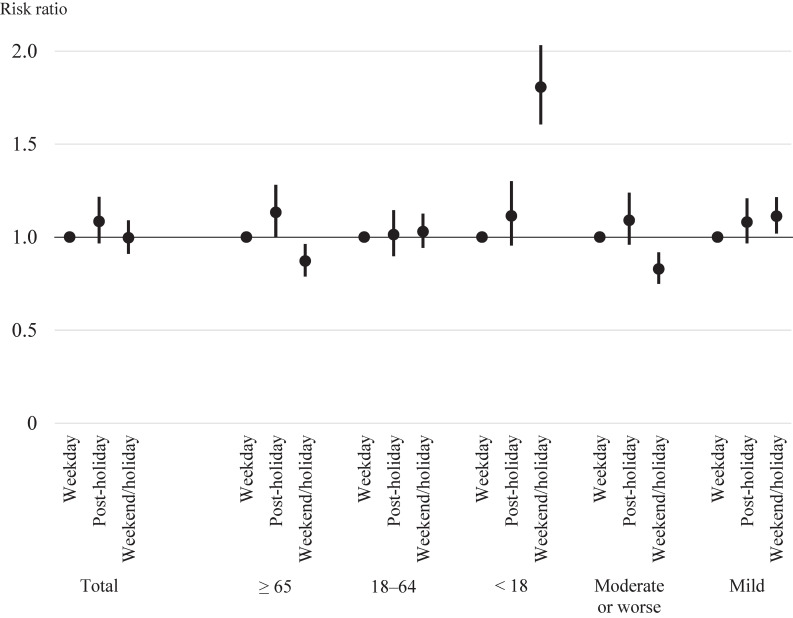
Risk ratios of heatstroke-related ambulance transports according to day type Points represent risk ratios and error bars indicate 95% confidence intervals from modified Poisson regression models adjusted for WBGT (lag 0 and 1) and year. WBGT was defined as the highest daily maximum WBGT across seven locations in Tokyo. Day types were defined as post-holiday (Mondays and the day following a public holiday), weekday, and weekend/holiday (Saturdays, Sundays, and public holidays).

After adjusting for WBGT category and calendar year, heatstroke-related ambulance transports differed by day type (Fig. 1). Overall, ambulance transports tended to be more frequent on post-holiday days than on weekdays (RR, 1.08; 95% CI, 0.97–1.22). Age-specific analyses revealed distinct patterns across age groups. Among adults aged ≥65 years, ambulance transports were more frequent on post-holiday days (1.13, 1.00–1.28) and less frequent on weekends and holidays (0.87, 0.79–0.96) compared with weekdays. In contrast, among individuals aged <18 years, ambulance transports were markedly more frequent on weekends and holidays (1.80, 1.61–2.03). Differences according to day type were relatively small among adults aged 18–64 years. Patterns also varied according to severity. Mild cases were more frequent on weekends and holidays than on weekdays (1.11, 1.02–1.21), whereas moderate or more severe cases were less frequent on weekends and holidays (0.83, 0.75–0.92).

**Fig. 2:**
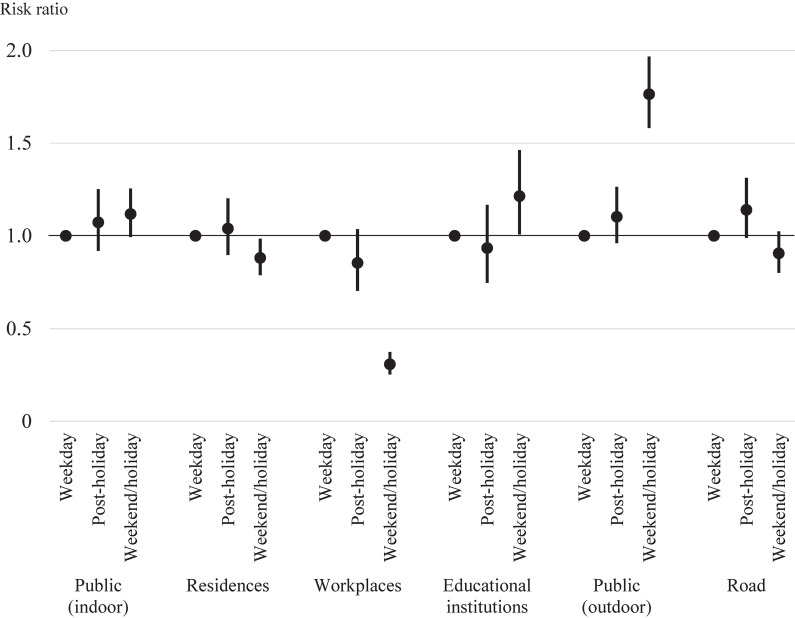
Risk ratios of heatstroke-related ambulance transports according to day type for each location of occurrence Points represent risk ratios and error bars indicate 95% confidence intervals from modified Poisson regression models adjusted for WBGT (lag 0 and 1) and year. WBGT was defined as the highest daily maximum WBGT across seven locations in Tokyo. Day types were defined as post-holiday (Mondays and the day following a public holiday), weekday, and weekend/holiday (Saturdays, Sundays, and public holidays). Locations of occurrence were categorised as public indoor spaces, residences, workplaces, educational institutions, public outdoor spaces, and roads.

Patterns also varied according to place of occurrence (Fig. 2). Heatstroke cases were more frequent on weekends and holidays in public indoor spaces (1.12, 0.99–1.26), public outdoor spaces (1.76, 1.58–1.97), and educational institutions (1.21, 1.01–1.46). In contrast, heatstroke cases were less frequent on weekends and holidays in residences (0.88, 0.79–0.98) and workplaces (0.31, 0.25–0.37). Cases on roads tended to be more frequent on post-holiday days, although not statistically significant.

In the sensitivity analyses, the results using the mean of the daily maximum WBGT across locations were largely consistent with the main findings (Supplementary Figs. 1 and 2). Similar results were also obtained when air temperature was used instead of WBGT (Supplementary Figs. 3 and 4) and when WBGT was modelled as a continuous variable (Supplementary Figs. 5 and 6). Analyses excluding 2020–2022 yielded broadly similar results, although the RRs tended to be slightly closer to unity (Supplementary Figs. 7 and 8).

## Discussion

In this time-series analysis of heatstroke-related ambulance transports in Tokyo, we provided the first evidence that heatstroke occurrence differed according to day type, even after adjusting for WBGT. Distinct patterns were observed across age groups, severity, and place of occurrence. In particular, ambulance transports among adults aged ≥65 years were more frequent on post-holiday days and less frequent on weekends, whereas transports among individuals aged <18 years were markedly more frequent on weekends and holidays.

Differences according to day type persisted even after adjusting for WBGT. This suggests that meteorological conditions alone cannot fully explain temporal variations in heatstroke occurrence. Therefore, behavioural factors related to weekly activity patterns may play an important role. Activities, such as work, sports, commuting, and recreation, often differ systematically between weekdays and weekends or holidays, potentially modifying the heatstroke risk even under similar heat conditions.

Among older adults, ambulance transports were more frequent on post-holiday days. Similar patterns, in which emergency visits or mortality increases at the beginning of the week, particularly for cardiovascular conditions, have been reported in previous studies [17–19]. This may be explained by several factors, including delayed medical care due to limited access on weekends. Behavioural and physiological factors may also contribute. The number of older workers in Japan has increased in recent years [20]. Resumption of work and routine activities at the beginning of the week may increase heat exposure among some older adults. A temporary reduction in heat acclimatisation after a holiday may also influence susceptibility to heat-related illness. A previous study in Japan also reported that ambulance transport for workplace-related heatstroke was significantly more frequent on Mondays among women [21]. However, that study did not adjust for meteorological conditions, such as WBGT. Because the present study lacked information on occupation, activity patterns, and heat acclimatisation, the role of temporary reductions in heat acclimatisation remains hypothetical. In contrast, ambulance transports were more frequent among those aged <18 years on weekends and holidays, consistent with a previous study showing that most paediatric heat-related illnesses are associated with sports or outdoor activities [22]. Therefore, increased participation in such activities on weekends may have contributed to this pattern.

Findings according to the severity and place of occurrence also support the importance of behavioural factors. Mild cases were more frequent on weekends and holidays, whereas severe cases were less frequent. This may reflect an increase in activity-related (exertional) heatstroke during weekends, along with a decrease in cases associated with older adults, who are more likely to develop severe heat-related illnesses. Consistent with this interpretation, heatstroke in residential areas occurred less frequently on weekends and holidays. Because heatstroke occurring in residences is more common among older adults, the reduction during weekends may reflect increased opportunities for family members and others to supervise them, which may also contribute to fewer severe cases. In contrast, increased participation in outdoor sports and leisure activities on weekends may explain the higher frequency of cases in public spaces and educational institutions.

This study has some limitations. First, heat exposure was represented using WBGT data from seven locations and may not fully capture local variation. However, WBGT values were highly correlated across locations and not dominated by one location (Supplementary Tables 1 and 2). Second, this study was based on aggregated data and lacked individual-level information (e.g. sex, occupation, pre-existing medical conditions, housing conditions, and air-conditioning use), limiting insight into mechanisms underlying differences by day type. Third, the recorded place of occurrence refers to where ambulance transport was initiated and may not reflect the actual location of symptom onset. Therefore, some misclassification of the place of occurrence may have occurred, and place-specific findings should be interpreted with caution. Fourth, as this study focused on Tokyo, caution is required when generalising the findings to other regions with different urban structures, population demographics, and lifestyles.

In conclusion, heatstroke-related ambulance transports in Tokyo varied according to day type even after adjusting for WBGT. Ambulance transports were more frequent on post-holiday days among older adults and on weekends and holidays among individuals aged <18 years. Weekend and holiday increases were also observed for mild cases and for cases occurring in public indoor and outdoor spaces and educational institutions.

## Abbreviations

WBGT: wet bulb globe temperature
RR: risk ratio
CI: confidence interval
COVID-19: coronavirus disease 2019

## Supplementary information

**Additional file 1:** **Supplementary Figure 1.** Risk ratios of heatstroke-related ambulance transports according to day type using the average of daily maximum WBGT. **Supplementary Figure 2.** Risk ratios of heatstroke-related ambulance transports according to day type for each location of occurrence using the average of daily maximum WBGT. **Supplementary Figure 3.** Risk ratios of heatstroke-related ambulance transports according to day type using air temperature instead of WBGT. **Supplementary Figure 4.** Risk ratios of heatstroke-related ambulance transports according to day type for each location of occurrence, using air temperature instead of WBGT. **Supplementary Figure 5.** Risk ratios of heatstroke-related ambulance transports according to day type using WBGT as continuous variables. **Supplementary Figure 6.** Risk ratios of heatstroke-related ambulance transports according to day type for each location of occurrence using WBGT as continuous variables. **Supplementary Figure 7.** Risk ratios of heatstroke-related ambulance transports according to day type after excluding 2020–2022. **Supplementary Figure 8.** Risk ratios of heatstroke-related ambulance transports according to day type for each location of occurrence after excluding 2020–2022. **Supplementary Table S1.** Correlation Matrix of Daily Maximum WBGT Values Across the Seven Locations. **Supplementary Table S2.** Distribution of Locations with the Highest Daily Maximum WBGT.

## Declarations

### Availability of data and materials

Data are derived from a source in the public domain (https://www.fdma.go.jp/disaster/heatstroke/post4.html.).
