# Supplementary material for: Day type and heatstroke-related ambulance transports in Tokyo, Japan: a time-series study
Source: Environ Health Prev Med. 2026 Aug 7;31:51. doi: 10.1265/ehpm.26-00077 (PMC13482159; doi:10.1265/ehpm.26-00077)
Supplement: Supplementary file 1 [file ehpm-31-051-s001.pdf]

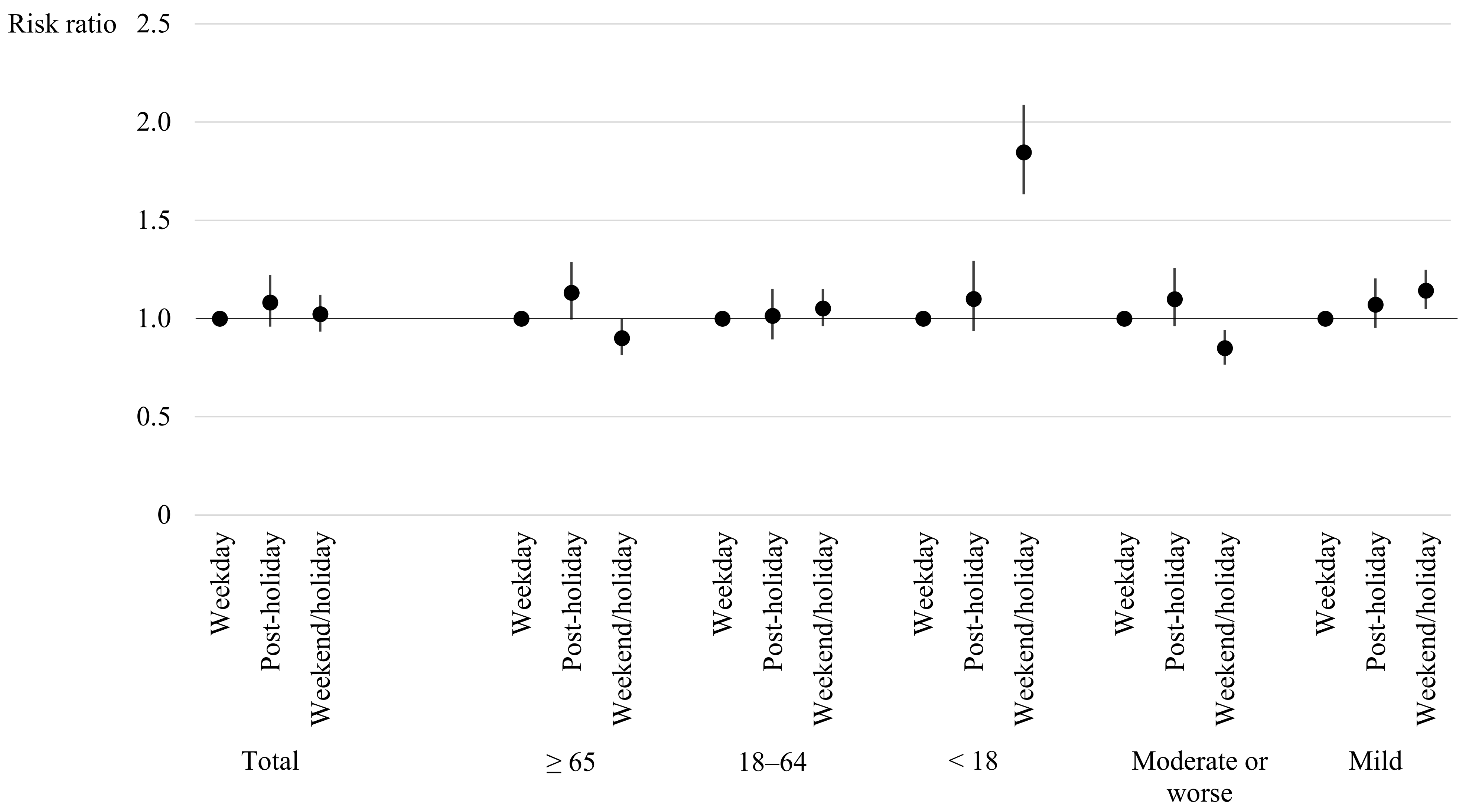

Supplementary Figure 1. Risk ratios of heatstroke-related ambulance transports according to day type using the average of daily maximum WBGT

Points represent risk ratio and error bars indicate 95% confidence intervals from modified Poisson regression models adjusted for WBGT (lag 0 and 1) and year. WBGT was defined as the average of daily maximum WBGT across seven locations in Tokyo. Day types were defined as post-holiday (Mondays and the day following a public holiday), weekday, and weekend/holiday (Saturdays, Sundays, and public holidays).

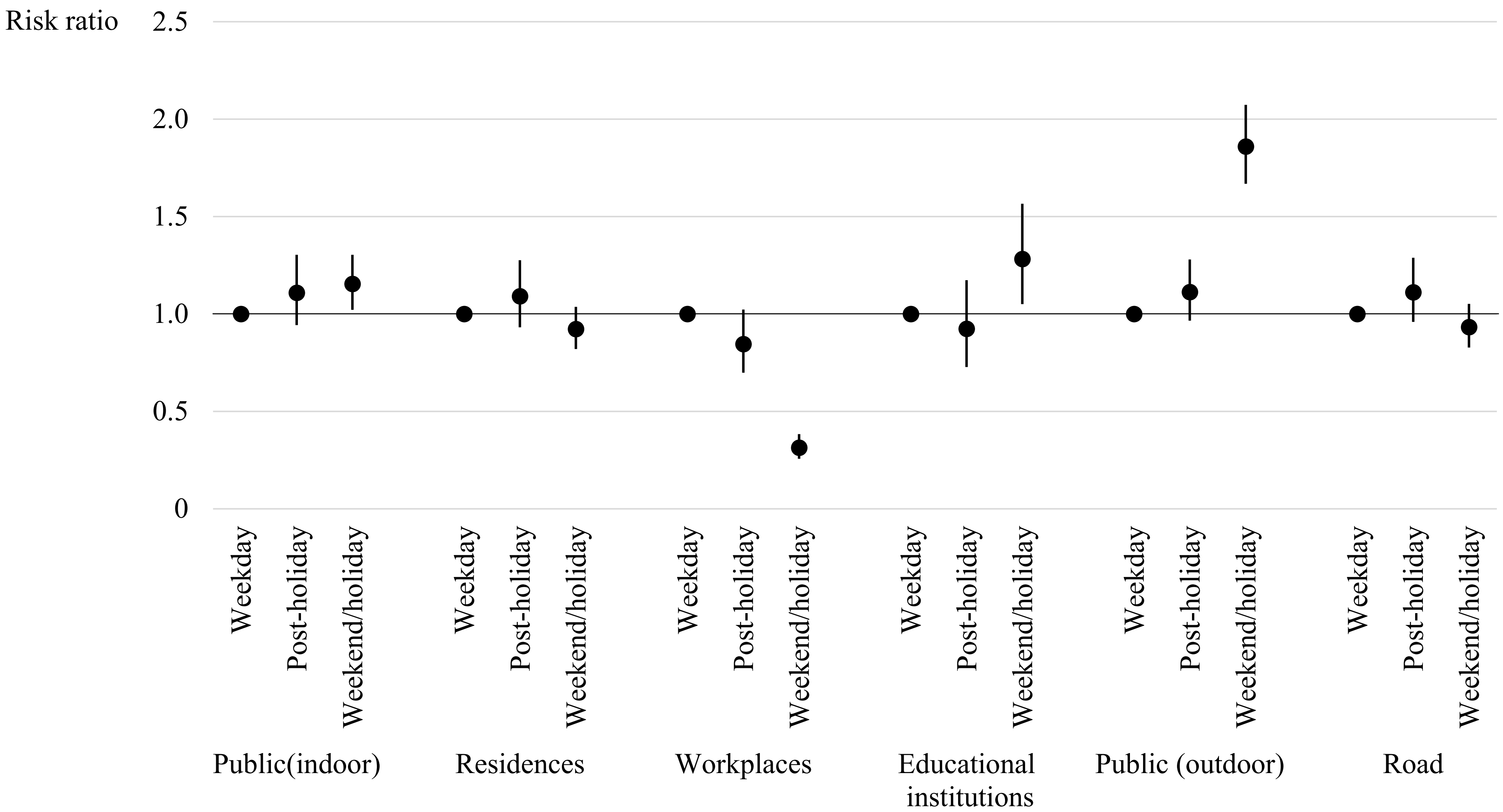

Supplementary Figure 2. Risk ratios of heatstroke-related ambulance transports according to day type for each location of occurrence using the average of daily maximum WBGT

Points represent risk ratios and error bars indicate 95% confidence intervals from modified Poisson regression models adjusted for WBGT (lag 0 and 1) and year. WBGT was defined as the average of daily maximum WBGT across seven locations in Tokyo. Day types were defined as post-holiday (Mondays and the day following a public holiday), weekday, and weekend/holiday (Saturdays, Sundays, and public holidays). Locations of occurrence were categorized as public indoor spaces, residences, workplaces, educational institutions, public outdoor spaces, and roads.

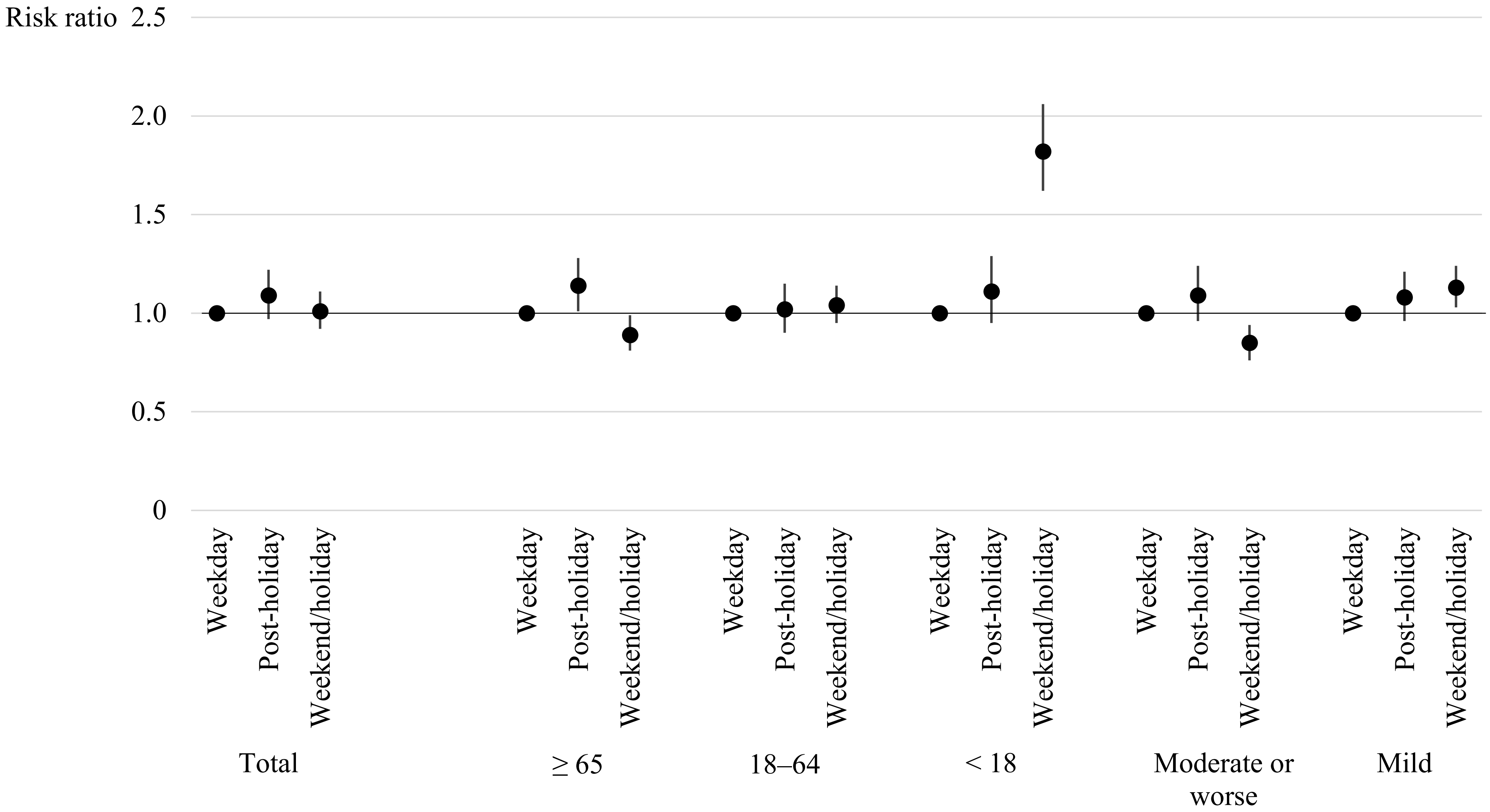

Supplementary Figure 3. Risk ratios of heatstroke-related ambulance transports according to day type using air temperature instead of WBGT

Points represent risk ratios and error bars indicate 95% confidence intervals from modified Poisson regression models adjusted for air temperature and year.
Air temperature was defined using daily mean air temperature data from the monitoring site in Kitanomaru, Tokyo. Day types were defined as post-holiday (Mondays and the day following a public holiday), weekday, and weekend/holiday (Saturdays, Sundays, and public holidays).

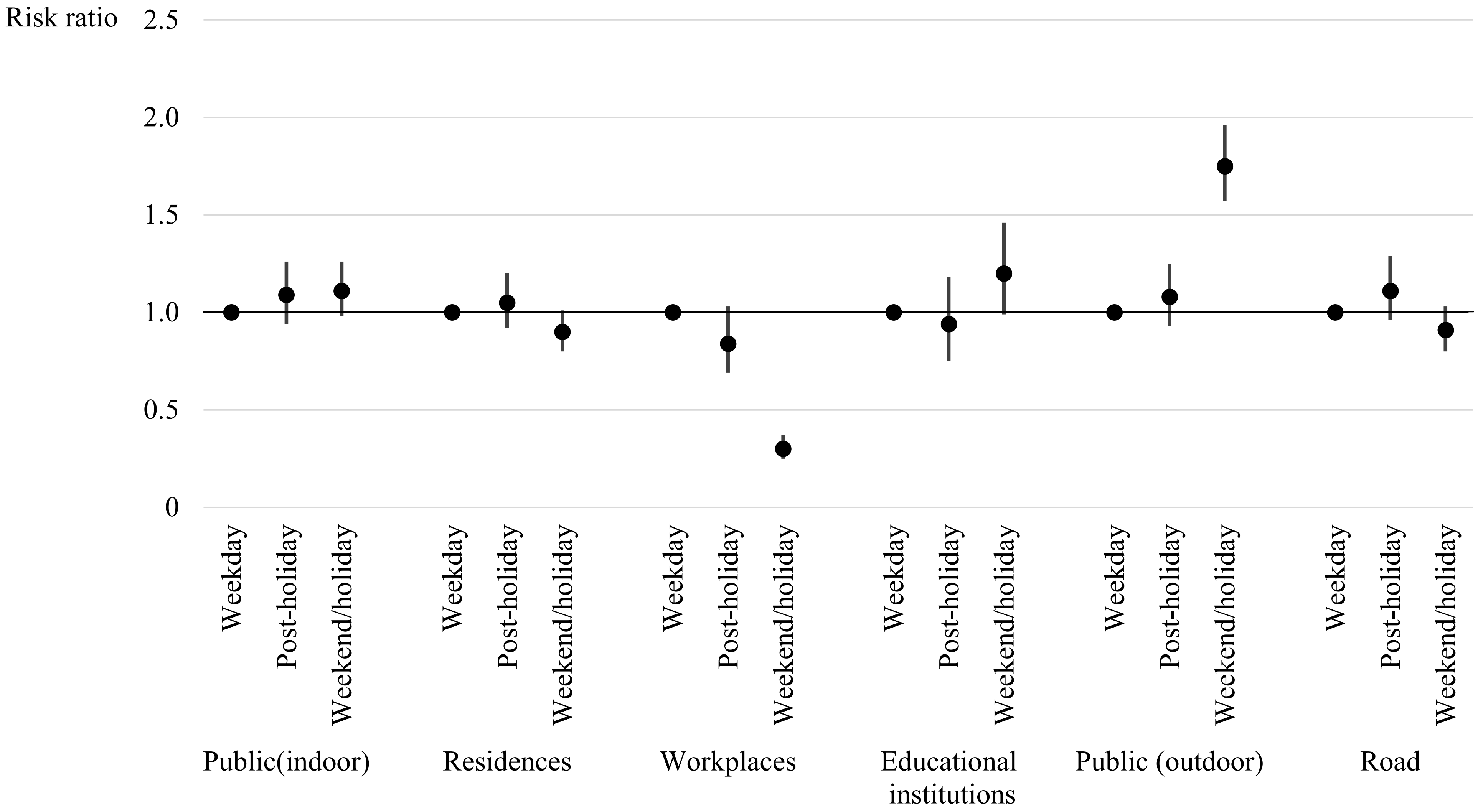

Supplementary Figure 4. Risk ratios of heatstroke-related ambulance transports according to day type for each location of occurrence, using air temperature instead of WBGT

Points represent risk ratios and error bars indicate 95% confidence intervals from modified Poisson regression models adjusted for air temperature and year.
Air temperature was defined using daily mean air temperature data from the monitoring site in Kitanomaru, Tokyo. Day types were defined as post-holiday (Mondays and the day following a public holiday), weekday, and weekend/holiday (Saturdays, Sundays, and public holidays). Locations of occurrence were categorized as public indoor spaces, residences, workplaces, educational institutions, public outdoor spaces, and roads.

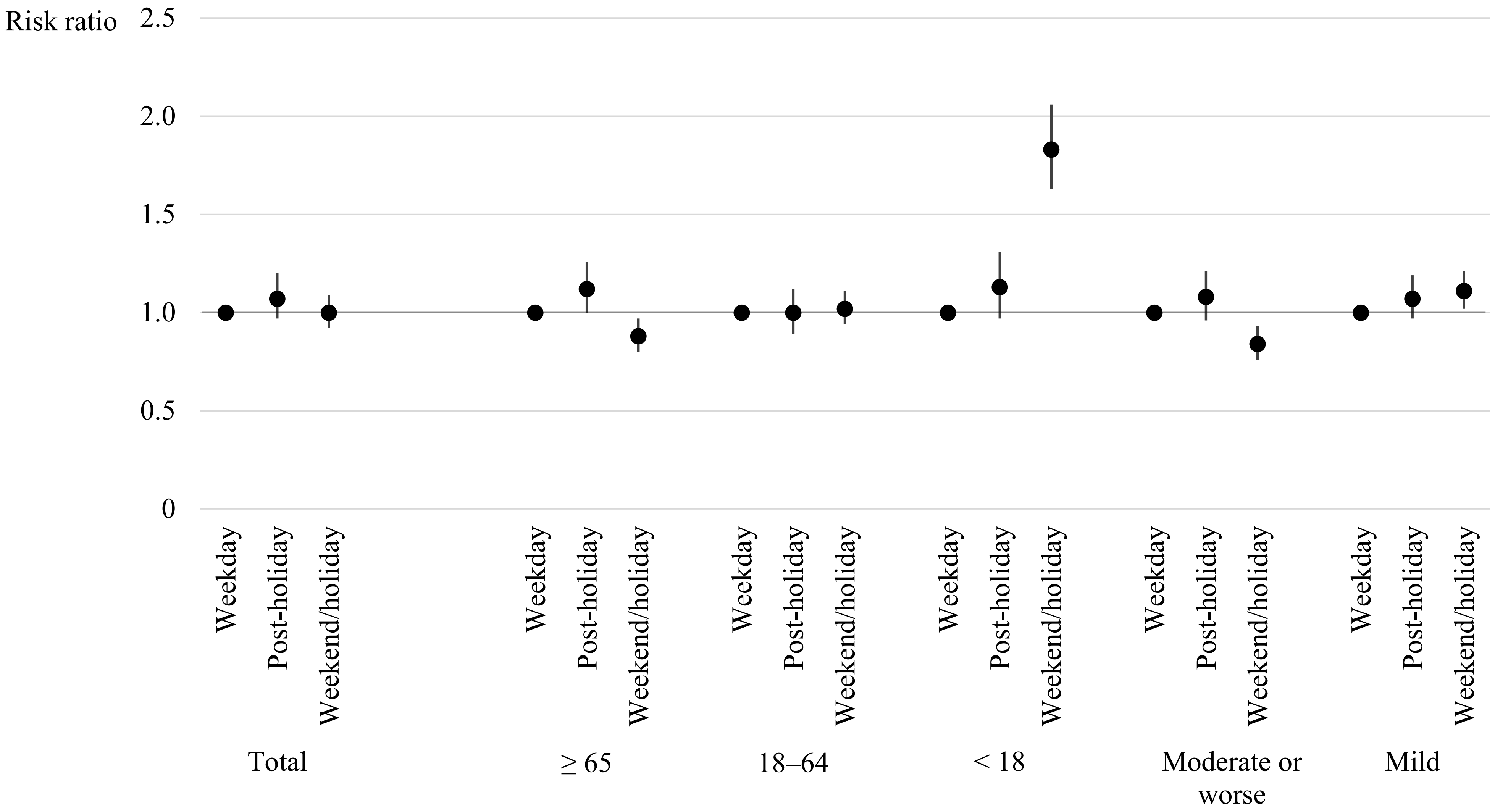

Supplementary Figure 5. Risk ratios of heatstroke-related ambulance transports according to day type using WBGT as continuous variables

Points represent risk ratios and error bars indicate 95% confidence intervals from modified Poisson regression models adjusted for WBGT (lag 0 and 1) and year. WBGT was included as continuous variables, using the highest daily maximum WBGT across seven locations in Tokyo. Day types were defined as post-holiday (Mondays and the day following a public holiday), weekday, and weekend/holiday (Saturdays, Sundays, and public holidays).

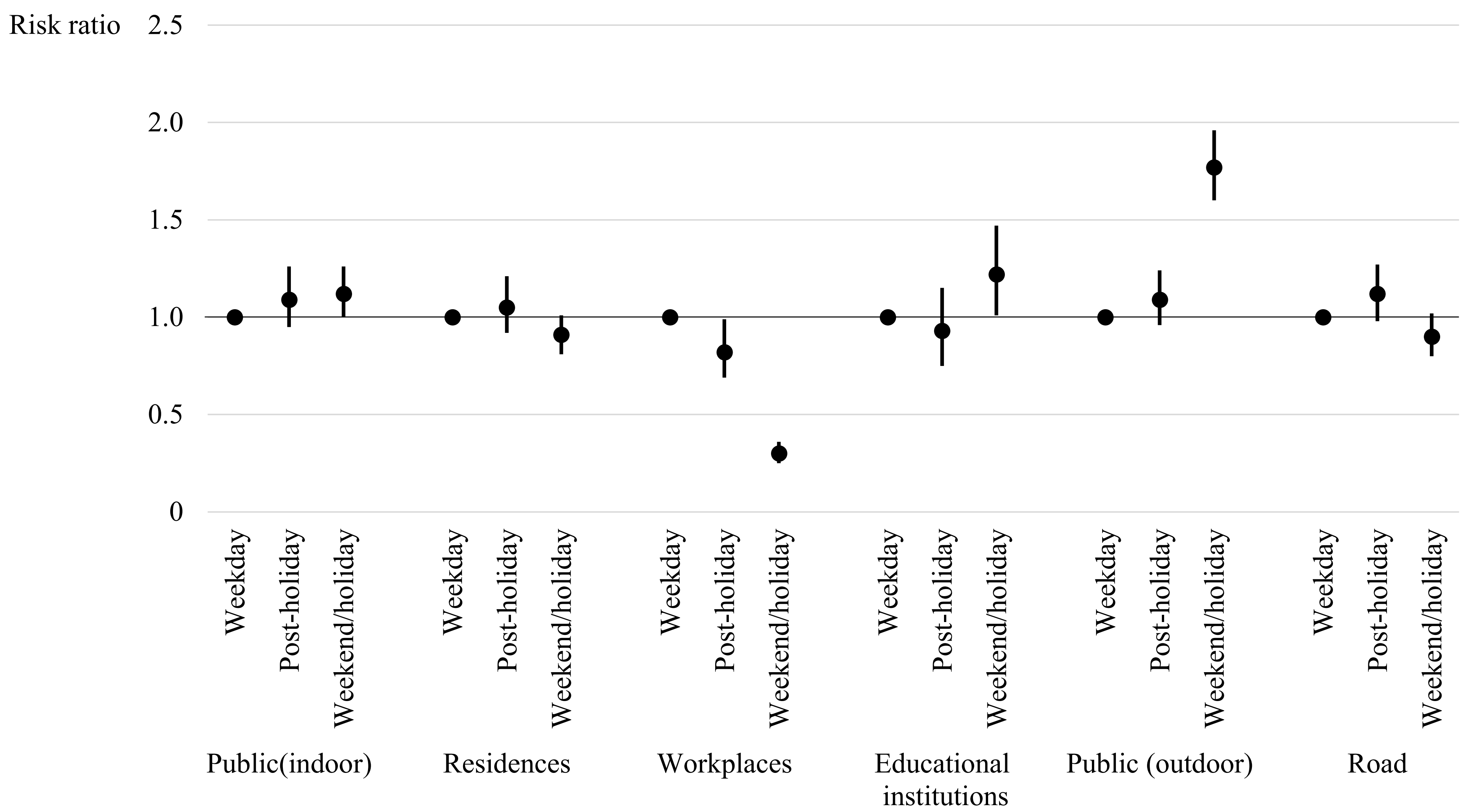

Supplementary Figure 6. Risk ratios of heatstroke-related ambulance transports according to day type for each location of occurrence using WBGT as continuous variables

Points represent risk ratios and error bars indicate 95% confidence intervals from modified Poisson regression models adjusted for WBGT (lag 0 and 1) and year. WBGT was included as continuous variables, using the highest daily maximum WBGT across seven locations in Tokyo. Day types were defined as post-holiday (Mondays and the day following a public holiday), weekday, and weekend/holiday (Saturdays, Sundays, and public holidays). Locations of occurrence were categorized as public indoor spaces, residences, workplaces, educational institutions, public outdoor spaces, and roads.

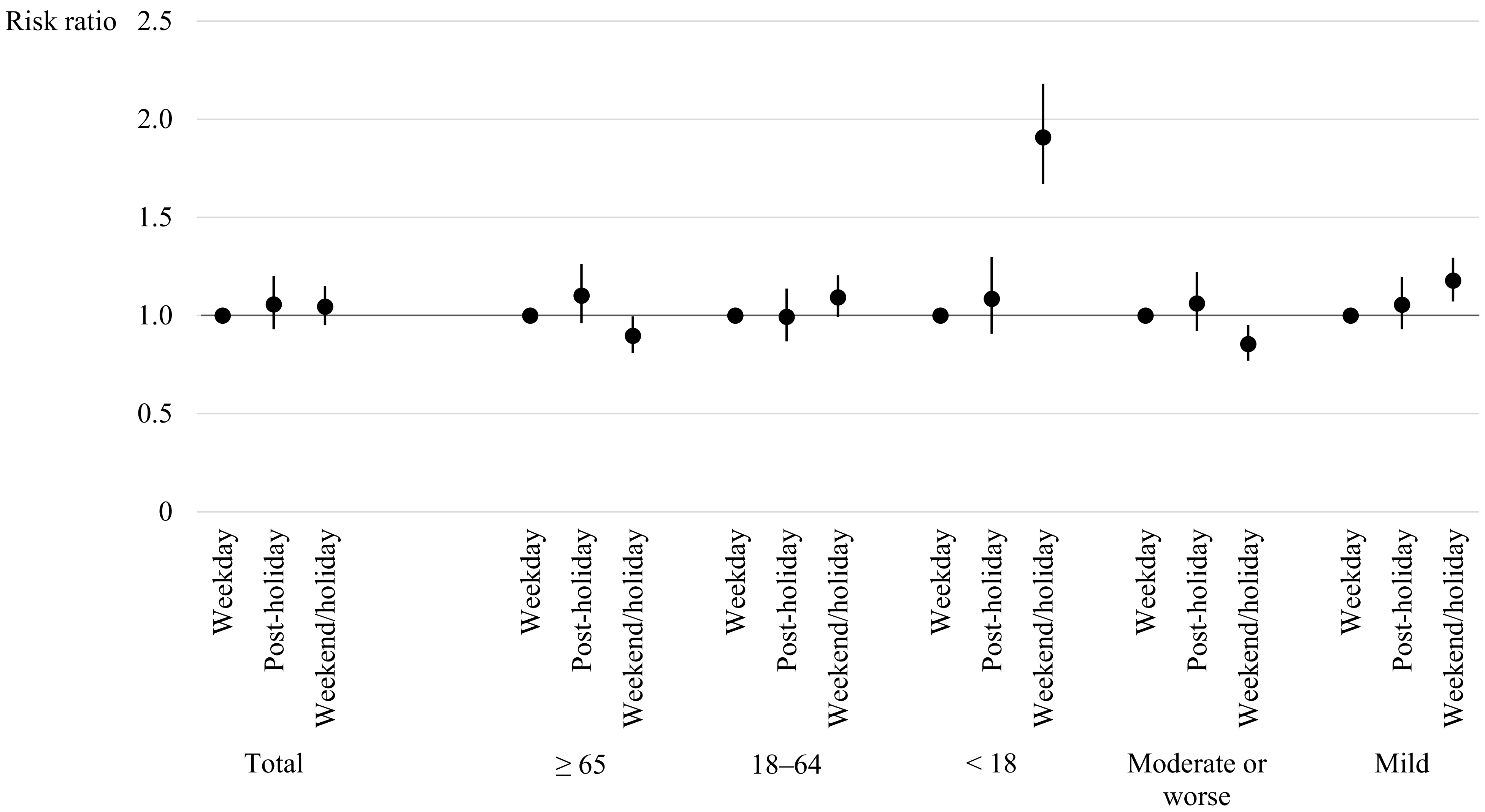

Supplementary Figure 7. Risk ratios of heatstroke-related ambulance transports according to day type after excluding 2020–2022

Points represent risk ratio and error bars indicate 95% confidence intervals from modified Poisson regression models adjusted for WBGT (lag 0 and 1) and year. Data from 2020–2022 were excluded to minimize the potential impact of the COVID-19 pandemic. WBGT was defined as the highest daily maximum WBGT across seven locations in Tokyo. Day types were defined as post-holiday (Mondays and the day following a public holiday), weekday, and weekend/holiday (Saturdays, Sundays, and public holidays).

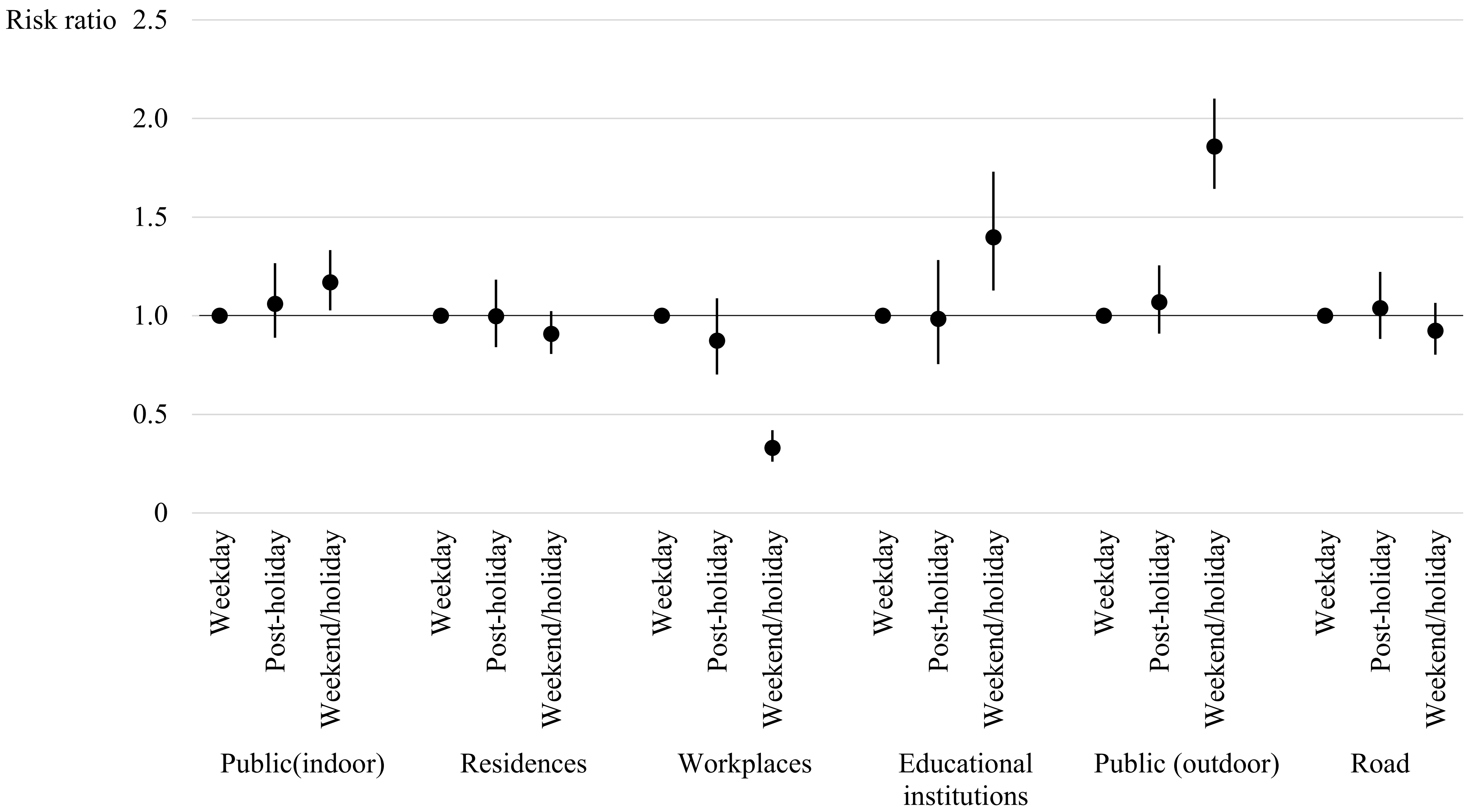

Supplementary Figure 8. Risk ratios of heatstroke-related ambulance transports according to day type for each location of occurrence after excluding 2020–2022

Points represent risk ratios and error bars indicate 95% confidence intervals from modified Poisson regression models adjusted for WBGT (lag 0 and 1) and year. Data from 2020–2022 were excluded to minimize the potential impact of the COVID-19 pandemic. WBGT was defined as the highest daily maximum WBGT across seven locations in Tokyo. Day types were defined as post-holiday (Mondays and the day following a public holiday), weekday, and weekend/holiday (Saturdays, Sundays, and public holidays). Locations of occurrence were categorized as public indoor spaces, residences, workplaces, educational institutions, public outdoor spaces, and roads.

Supplementary Table S1. Correlation Matrix of Daily Maximum WBGT Values Across the Seven Locations

	Bunkyo	Nerima	Hachioji	Fuchu	Edogawa-Rinkai	Ogouchi	Ome
Bunkyo	1						
Nerima	0.973	1					
Hachioji	0.970	0.977	1				
Fuchu	0.974	0.985	0.983	1			
Edogawa-Rinkai	0.938	0.930	0.923	0.939	1		
Ogouchi	0.952	0.950	0.960	0.956	0.904	1	
Ome	0.967	0.977	0.985	0.980	0.921	0.961	1

Supplementary Table S2.
Distribution of Locations with the Highest Daily Maximum WBGT

	n(%)
Bunkyo	311 (18.8)
Nerima	494 (29.9)
Hachioji	213 (12.9)
Fuchu	137 (8.3)
Edogawa-Rinkai	319 (19.3)
Ogouchi	10 (0.6)
Ome	168 (10.2)
